# Risk of second primary Cancer among bladder Cancer patients: a population-based cohort study in Korea

**DOI:** 10.1186/s12885-018-4530-3

**Published:** 2018-05-31

**Authors:** Whi-An Kwon, Jae Young Joung, Jiwon Lim, Chang-Mo Oh, Kyu-Won Jung, Sung Han Kim, Ho Kyung Seo, Weon Seo Park, Jinsoo Chung, Kang Hyun Lee, Young-Joo Won

**Affiliations:** 1 Center for Prostate Cancer, National Cancer Center, Goyang, Korea; 2 Cancer Registration and Statistics Branch, National Cancer Center, Goyang, Korea

**Keywords:** Bladder cancer, Second primary cancer, Prognosis, Incidence, Survival

## Abstract

**Background:**

For the expanding population of bladder cancer survivors in Korea, the development of subsequent cancers is a significant concern. Here, we provide the second primary cancer incidence rates and types in Korean patients with bladder cancer.

**Methods:**

Using population-based data from the Korea Central Cancer Registry from 1993 to 2013, we studied the standardized incidence ratios among 48,875 individuals with an initial diagnosis of bladder cancer. Standardized incidence ratios for second primary cancers were evaluated according to age at diagnosis, latency, diagnostic year, and treatment.

**Results:**

Over the same period, the overall risk of a second primary cancer was reduced by 6% in patients with bladder cancer compared with the development of a new malignancy in the general population (standardized incidence ratio = 0.94; 95% CI, 0.91–0.97, *p* < 0.05). For specific cancers, the standardized incidence ratios for stomach, colon, liver, and non-Hodgkin lymphoma were significantly lower in patients with bladder cancer. However, the risk of prostate and kidney cancer in patients with bladder cancer were significantly increased. The risk of lung squamous cell carcinoma and lung adenocarcinoma as second primary cancers was significantly elevated in patients with bladder cancer.

**Conclusion:**

Korean patients with bladder cancer have a 6% lower risk of developing a second primary cancer. However, they have a higher risk of developing subsequent prostate and kidney cancers, lung squamous cell carcinoma, and lung adenocarcinoma, suggesting the need for continual intensive cancer surveillance among bladder cancer survivors.

**Electronic supplementary material:**

The online version of this article (10.1186/s12885-018-4530-3) contains supplementary material, which is available to authorized users.

## Background

Bladder cancer (BC) is the 9th most frequent cancer worldwide [1] and the number of BC cases increased from 2180 in 1999 to 3549 in 2011, with 37,950 total cases during this period in Korea [2]. Moreover, according to the Korea Central Cancer Registry (KCCR) report, 3949 new BC cases were diagnosed in 2014, with 7.8 cases per 100,000 person-years [3].

There is a long-term survival concern in patients with BC, especially those with second primary cancer (SPC). For Western patients, compared with the general population, BC survivors are more likely to develop SPCs, which frequently occur in the lungs or neck [4, 5]. However, to our knowledge, no studies have evaluated SPC among Asian patients with BC. Although, we have previously detailed the overall risk of SPC development in Korean patients with prostate cancer and kidney cancer [6, 7]. Therefore, we were also interested in studying SPC in patients with primary BC.

The purpose of this population-based cohort study was to calculate the incidence of SPC in Korean patients with BC and to estimate the effect of SPC on survival using a nationwide population-based cancer registry. The primary goal was to produce useful data for managing patients with BC.

## Methods

### Study population and data collection

A total of 48,875 patients diagnosed with BC were evaluated between 1993 and 2013 as documented in the KCCR. The KCCR gathers information on ~ 80–90% of cancer cases across 180 hospitals across South Korea. In 1999, the scope of the KCCR was expanded to cover the entire South Korean population using the Population-Based Cancer Registry Program [8].

To ensure that SPC remains distinct from primary BC recurrences and metastases, the KCCR uses coding rules based on the histological or topographical classifications of the International Classification of Diseases for Oncology 3rd edition [9] and the International Agency for Research on Cancer (IARC) rules for multiple primary cancer in 2004 [10]. The IARC classifies cancer as an SPC when a primary tumor has a different histological type or anatomical site from the indexed cancer. KCCR data includes patient information (age at the time of diagnosis and sex), cancer information (diagnosis date, tumor site, histology, and surveillance, epidemiology, and end results [SEER] summary stage), and primary treatment information (surgery, chemotherapy, or radiotherapy).

The first primary BC included patients with a single primary BC and the first BC in patients with multiple primary cancers. We excluded the following first primary BC cases: 1) age at diagnosis, unknown; and 2) BC reported at death. In addition, because SPCs diagnosed within two months of the first primary cancer diagnosis are considered synchronous, these cases were excluded to reduce the misclassification of undetected synchronous cancers and metastases.

Ethical approval for the research protocol was provided by the institutional review board of the National Cancer Center (NCC2017–0182).

### Statistical analyses

Standardized incidence ratios (SIR) were used to compare the relative risk of the SPC incidence rates with those of the general population at baseline. We estimated cancer incidence for each cancer type according to age at diagnosis, latency, and diagnostic year, which was multiplied by the cumulative number of years at risk to calculate the number of cancer outbreaks expected for each stratum. SIR was estimated by dividing the observed number of SPCs in patients with BC by the number of patients at risk of developing a new malignancy in the general population. The 95% confidence intervals for the SIRs were estimated using Byar’s exact approximation to the accurate Poisson distribution of the observed number. The person-years at risk were calculated from two months after the initial BC diagnosis until death, the date of last known survival, or the study completion date (December 31st, 2013).

Results were classified based on age at the time of initial diagnosis with BC (0–39, 40–59, or ≥ 60 years), year of first BC diagnosis (1993–2000 or 2001–2013), latency time among first BC diagnosis and subsequent primary cancer (< 12 months, 12–59 months, 60–119 months, or ≥ 120 months), and treatment type (surgery vs. non-surgery, chemotherapy vs. non-chemotherapy, and radiotherapy [RT] vs. non-RT).

Survival curves using the Kaplan-Meier method were calculated for BC patients with or without a subsequent cancer. The log-rank test was employed to verify the difference between groups of survival curves. All of the statistical tests were determined statistically significant at *P*-value < 0.05, and were two-sided. The SIR and 95% CI calculations were performed using SEER*Stat (seer.cancer.gov/seerstat, version 8.3.4). Survival analyses and log-rank tests were performed using STATA (StataCorp LP, version 12.1).

## Results

We obtained data from 48,875 patients, including 39,351 men (80.5%) and 9524 women (19.5%), with a median age at diagnosis of 67 years. The cohort characteristics are shown in Table 1. The overall SPC risk decreased by 6% in patients with previous BC compared with that in the general population over the same period (SIR = 0.94; 95% CI, 0.91–0.97). Patients examined within one year of BC diagnosis exhibited an increased risk of all subsequent cancers (SIR = 1.21). Patients who were followed up for 1–5 years showed a SIR risk reduction of 0.89. Finally, after a ≥ 10-year follow-up, the SIR decreased to 0.86. Patients aged < 40 years at BC diagnosis were more likely to have all SPC types (SIR = 1.50); whereas, those aged 40–59 or ≥ 60 years at diagnosis exhibited a reduced SPC incidence (SIR = 1.04 and 0.90, respectively). Two periods were analyzed (1993–2000 and 2001–2013) to evaluate the potential impact of changes in diagnosis and treatment. SPC incidence differed between these two periods (SIR = 0.85 and 0.99, respectively; Table 2).

**Table 1:** Characteristics of patients with primary BC, 1993–2013

	Total		Men		Women	
	n	%	n	%	n	%
Patients with BC	48,875	100	39,351	100	9524	100
Period of BC diagnosis						
1993–1997	6892	14.10	5592	14.21	1300	13.65
1998–2002	10,484	21.45	8377	21.29	2107	22.12
2003–2007	13,585	27.80	10,942	27.81	2643	27.75
2008–2013	17,914	36.65	14,440	36.70	3474	36.48
Average age at diagnosis with BC (years; mean, SD)	65.39	12.46	64.81	12.20	67.80	13.22
Median age at diagnosis with BC (years; median, range)	67	105 (1–106)	66	100 (1–101)	70	105 (1–106)
Age at primary BC diagnosis (years)						
0–39	1608	3.29	1259	3.20	349	3.66
40–59	12,489	25.55	10,601	26.94	1888	19.82
≥60	34,778	71.16	27,491	69.86	7287	76.51
Percentage of primary treatment status						
Surgery	42,448	86.85	34,653	88.06	7795	81.85
Radiation	1298	2.66	1004	2.55	294	3.09
Chemotherapy	6161	12.61	5045	12.82	1116	11.72
Average follow-up after BC diagnosis (years; mean, SD)	5.65	5.09	5.70	5.06	5.46	5.21
Median follow-up after BC diagnosis (years; median, range)	4.13	20.80(0–20.80)	4.21	20.80(0–20.80)	3.67	20.80(0–20.80)
Number of patients who developed a SPC	3495	7.15	3116	7.92	379	3.98
Average age at SPC diagnosis (years; mean, SD)	70.57	9.28	70.71	8.94	69.40	11.67
Median age at SPC diagnosis (years; median, range)		71(10–95)		71.5(10–95)		71(12–93)
Average interval between primary cancer and SPC (years; mean, SD)	5.23	4.30	5.18	4.29	5.75	4.39
Median interval between primary cancer and SPC (years; median, range)		4.17(0.17–0.67)		4.08(0.17–20.67)		4.75(0.17–19.08)
Number of patients by latency between primary cancer and SPC (years)						
1	556	15.91	469	12.95	47	16.19
1–4	1409	40.31	1176	39.67	144	40.61
5–9	1000	28.61	813	29.20	106	28.07
≥10	530	15.16	438	18.18	66	15.12
Number of patients by age at SPC diagnosis (years)						
0–39	13	0.37	8	0.26	5	1.32
40–59	396	11.33	327	10.49	69	18.21
≥60	3086	88.30	2781	89.25	305	80.47
Average follow-up after SPC diagnosis, (years; mean, SD)	2.55	2.91	2.48	2.82	3.10	3.52
Median follow-up after SPC diagnosis (years; median, range)	1.42	20.00(0–20.00)	1.42	20.00(0–20.00)	1.67	19.50(0–19.50)
Number of subsequent primary cancers						
1	3259	6.67	2896	7.36	363	3.81
2	217	0.44	203	0.52	14	0.15
≥3	19	0.04	17	0.04	2	0.02

*BC*: bladder cancer, *SD*: standard deviation, *SPC*: second primary cancer

**Table 2:** Risk of SPC after BC diagnosis by follow-up, age, and period (1993–2013)

	Total			latency (months)				Age (years)			Period	
				< 12	12–59	60–119	≥120	0–39	40–59	≥ 60	1993–2000	2001–2013
	SIR	O/E	CI	SIR	SIR	SIR	SIR	SIR	SIR	SIR	SIR	SIR
All SPCs	0.94#	(3821/4086.59)	(0.91–0.97)	1.21#	0.89#	0.92#	0.86#	1.50#	1.04	0.90#	0.85#	0.99
All SPCs excluding BC, KC, pelvic and ureteral cancer	0.96#	(3751/3921.87)	(0.93–0.99)	1.20#	0.91#	0.95	0.90#	1.36	1.06	0.92#	0.87#	1.01
Buccal cavity, pharynx	0.79	(53/67.09)	(0.59–1.03)	0.23#	1.09	0.62	0.76	0	1.03	0.71#	0.68	0.87
Tongue	0.37#	(4/10.95)	(0.10–0.94)	0	0.43	0.32	0.57	0	0.67	0.26#	0.22	0.47
Salivary gland	0.92	(6/6.51)	(0.34–2.01)	0	1.10	1.58	0	0	1.17	0.85	0.76	1.03
Tonsil	0.27#	(2/7.32)	(0.03–0.99)	0	0.66	0	0	0	0.40	0.21	0	0.44
Hypopharynx	1.19	(19/15.94)	(0.72–1.86)	0.49	1.33	0.86	2.01	0	1.69	1.05	1.48	0.98
Digestive system	0.85#	(1833/2160.13)	(0.81–0.89)	0.81#	0.79#	0.96	0.82#	1.41	0.95	0.81#	0.79#	0.89#
Esophagus	0.97	(94/97.04)	(0.78–1.19)	0.96	0.96	1.07	0.80	0	1.05	0.95	0.83	1.08
Stomach	0.79#	(632/796.08)	(0.73–0.86)	0.73#	0.73#	0.87#	0.87	1.47	0.92	0.75#	0.73#	0.84#
Small intestine	1.43	(20/14.02)	(0.87–2.20)	2.31	1.55	1.22	0.84	0	1.33	1.47	2.13#	0.95
Colon	0.84#	(271/321.74)	(0.74–0.95)	0.95	0.87	0.86	0.70#	1.72	0.90	0.82#	0.77#	0.88
Rectum, rectosigmoid junction	0.91	(233/255.77)	(0.80–1.04)	1.15	0.75#	1.02	0.95	1.59	0.88	0.91	0.82	0.96
Anus, anal canal	0.97	(5/5.15)	(0.32–2.27)	1.51	0.46	1.34	1.20	0	2.12	0.72	0.89	1.03
Liver	0.79#	(240/304.17)	(0.69–0.90)	0.63#	0.74#	0.94	0.79	1.09	0.95	0.71#	0.77#	0.80#
Gallbladder	0.77	(43/55.52)	(0.56–1.04)	1.18	0.57#	0.86	0.83	0	0.92	0.75	0.71	0.82
Bile ducts, other biliary	0.92	(166/180.66)	(0.78–1.07)	0.55#	0.92	1.18	0.73	2.04	0.95	0.91	0.92	0.92
Pancreas	0.98	(122/124.96)	(0.81–1.17)	0.67	0.98	1.19	0.82	2.2	1.27	0.90	0.81	1.08
Respiratory system	1.05	(848/807.86)	(0.98–1.12)	1.06	1.07	1.01	1.05	0	1.27#	1.01	0.93	1.13#
Nose, nasal cavity, ear	1.07	(8/7.51)	(0.46–2.10)	2.06	1.57	0.46	0	0	0	1.41	0.62	1.39
Larynx	1.20	(58/48.45)	(0.91–1.55)	2.18#	1.09	0.87	1.26	0	1.18	1.21	1.1	1.27
Lung, bronchus	1.05	(782/748.26)	(0.97–1.12)	0.98	1.07	1.03	1.06	0	1.30#	1	0.93	1.13#
Female breast	1.12	(34/30.38)	(0.78–1.56)	1.35	0.72	1.11	1.91	0.90	1.19	1.09	1.70#	0.78
Female genital system	1.10	(33/29.98)	(0.76–1.55)	1.71	1.16	0.59	1.33	2.75	0.97	1.07	1.27	0.96
Male genital system	1.45#	(513/353.32)	(1.33–1.58)	4.04#	1.30#	1.04	0.95	5.18	1.66#	1.41#	1.30#	1.53#
Prostate	1.46#	(505/346.97)	(1.33–1.59)	4.08#	1.31#	1.03	0.95	7.54	1.66#	1.41#	1.32#	1.53#
Testis	3.95	(3/0.76)	(0.82–11.55)	0	6.05	4.61	0	0	4.17	4.76	0	6.83#
Urinary system	0.73#	(170/232.70)	(0.62–0.85)	2.03#	0.72#	0.42#	0.37#	6.63#	0.89	0.65#	0.61#	0.81#
Urinary bladder	0.45#	(63/139.70)	(0.35–0.58)	1.63#	0.49#	0.12#	0.08#	12.41#	0.46#	0.41#	0.33#	0.54#
Kidney parenchyma	1.47#	(100/67.98)	(1.20–1.79)	3.23#	1.44#	1.04	1.08	4.54#	1.40	1.44#	1.32	1.56#
Renal pelvis, other urinary	0.28#	(7/25.02)	(0.11–0.58)	1.03	0.10#	0.40	0.00#	0	0.90	0.15#	0.43	0.19#
Brain, central nervous system	0.76	(16/21.09)	(0.43–1.23)	0.38	0.91	0.97	0.28	0	0.56	0.85	0.72	0.79
Thyroid	1.21	(107/88.29)	(0.99–1.46)	2.24#	1.29	0.97	0.83	1.08	1.35#	1.09	1.04	1.28#
Lymphatic, hematopoietic	0.75#	(98/130.52)	(0.61–0.92)	1.08	0.64#	0.73	0.83	0.63	0.57#	0.81	0.69#	0.79
Hodgkin lymphoma	0.77	(2/2.58)	(0.09–2.80)	3.11	0.92	0	0	0	0	1.15	0.99	0.64
Non-Hodgkin lymphoma	0.69#	(47/68.39)	(0.50–0.91)	1.21	0.75	0.35#	0.75	1.17	0.43#	0.76	0.64	0.71
Myeloma	0.82	(19/23.27)	(0.49–1.28)	1.09	0.64	0.87	0.95	0	0.79	0.83	0.70	0.89
Leukemia	0.82	(32/38.93)	(0.56–1.16)	0.84	0.44#	1.31	0.90	0	0.68	0.88	0.78	0.85

*SIR*: standardized incidence ratio, *CI:* confidence interval, *BC*: bladder cancer, *SPC*: second primary cancer, *KC*: kidney cancer, *O/E*: Observed/Expected,

# significant at alpha = 0.05

Significantly lower SIRs were observed for cancers of the tongue (SIR = 0.37; 95% CI 0.10–0.94), tonsil (SIR = 0.27; 95% CI 0.03–0.99), stomach (SIR = 0.79; 95% CI 0.73–0.86), colon (SIR = 0.84; 95% CI 0.74–0.95), and liver (SIR = 0.79; 95% CI 0.69–0.90), and for non-Hodgkin lymphoma (SIR = 0.69; 95% CI 0.50–0.91). However, the risks of prostate cancer and kidney cancer in patients with BC increased significantly (SIR = 1.46; 95% CI 1.33–1.59, and SIR = 1.47; 95% CI 1.20–1.79, respectively) (Table 2). Notably, SIR did not increase significantly for total lung cancers. However, a subgroup analysis based on lung cancer histology revealed that the SPC risks of lung squamous cell carcinoma and adenocarcinoma were significantly elevated (SIR = 1.15; 95% CI 1.02–1.29, and SIR = 1.20; 95% CI 1.05–1.37, respectively). By contrast, other lung cancer histologies (including small cell carcinoma: SIR = 1.06; 95% CI 0.86–1.28) were not associated with an increased SPC risk. Moreover, although not significant, the SIR increased after one year. SIRs for specific lung cancer types are shown in Table 3.

**Table 3:** Risk of SPC by lung cancer histology after BC diagnosis (1993–2013)

	Latency (months)					
	Total		< 12	12–59	60–119	≥120
	SIR (O/E)	95% CI	SIR (O/E)	SIR (O/E)	SIR (O/E)	SIR (O/E)
Lung, bronchus	1.05 (782/748.26)	(0.97–1.12)	0.98 (90/91.90)	1.07 (331/309.19)	1.03 (227/220.22)	1.06 (134/126.96)
Squamous cell carcinoma	1.15# (280/243.85)	(1.02–1.29)	0.94 (29/30.71)	1.18 (121/102.47)	1.08 (77/71.04)	1.34# (53/39.63)
Adenocarcinoma	1.20# (221/183.65)	(1.05–1.37)	1.39 (30/21.61)	1.27# (94/74.26)	1.15 (63/54.55)	1.02 (34/33.23)
Small cell carcinoma	1.06 (99/93.82)	(0.86–1.28)	0.69 (8/11.64)	1.10 (43/39.13)	1.13 (31/27.46)	1.09 (17/15.58)
Other and unspecified	0.80# (182/226.95)	(0.69–0.93)	0.82 (23/27.95)	0.78# (73/93.32)	0.83 (56/67.16)	0.78 (30/38.51)

*SPC*: second primary cancer, *BC*: bladder cancer, *SIR*: standardized incidence ratio, *CI*: confidence interval, *O/E*: Observed/Expected

# significant at alpha = 0.05

To estimate the effect of primary BC treatment on SPC risk, we calculated the SIR of the RT, surgery, and chemotherapy groups. For all treatment modalities except RT, the SPC risk was lower than that in comparable patients with BC. Effects of treatment on SPC risk are summarized in Table 4.

**Table 4:** Risk of SPC according to treatment for primary BC (1993–2013)

	RT	Non-RT	Surgery	Non-Surgery	Chemotherapy	Non-chemotherapy
	SIR	SIR	SIR	SIR	SIR	SIR
All SPCs	1.01	0.93#	0.94#	0.85#	0.94	0.93#
All SPCs excluding BC, KC, pelvic and ureteral cancers	1.04	0.96#	0.97#	0.86#	0.94	0.96#
Buccal cavity, pharynx	0	0.80	0.83	0.35	0.56	0.82
Tongue	0	0.37#	0.40	0	0.86	0.31#
Salivary gland	0	0.94	1.01	0	0	1.03
Tonsil	0	0.28	0.30	0	0	0.31
Hypopharynx	0	1.21	1.30	0	0.59	1.26
Digestive system	0.88	0.85#	0.87#	0.66#	0.78#	0.86#
Esophagus	0.60	0.98	0.97	0.95	0.86	0.98
Stomach	0.59	0.80#	0.81#	0.64#	0.71#	0.80#
Small intestine	4.18	1.38	1.49	0.79	0.68	1.51
Colon	1.35	0.83#	0.89#	0.39#	1.04	0.82#
Rectum, rectosigmoid junction	1.20	0.91	0.91	0.90	0.87	0.92
Anus, anal canal	0	0.99	0.86	1.98	1.86	0.87
Liver	0.98	0.79#	0.80#	0.70	0.73	0.80#
Gallbladder	0	0.79	0.78	0.74	0.17#	0.84
Bile ducts, other biliary	0.64	0.92	0.96	0.53	0.91	0.92
Pancreas	1.4	0.97	1.01	0.69	0.62	1.02
Respiratory system	1.16	1.05	1.05	1.01	0.98	1.06
Nose, nasal cavity, ear	7.75	0.95	1.17	0	1.25	1.04
Larynx	0	1.22	1.13	1.90	0.95	1.23
Lung, bronchus	1.18	1.04	1.05	0.97	0.98	1.05
Female breast	0	1.14	1.20	0.34	0.62	1.18
Female genital system	1.50	1.09	1.12	0.93	1.86	1.01
Male genital system	2.23#	1.44#	1.43#	1.74#	1.85#	1.41#
Prostate	2.28#	1.44#	1.43#	1.70#	1.83#	1.41#
Testis	0	4.02	4.32	0	12.33	2.95
Urinary system	1.03	0.73#	0.69#	1.1	1.28	0.67#
Urinary bladder	0	0.46#	0.43#	0.61	1.03	0.38#
Kidney parenchyma	2.81	1.45#	1.40#	2.29#	1.98#	1.41#
Renal pelvis, other urinary	2.43	0.24#	0.22#	0.90	0.78	0.22#
Brain, central nervous system	0	0.77	0.78	0.54	0	0.85
Thyroid	0	1.23#	1.23#	1	1.65	1.16
Lymphatic, hematopoietic	0.46	0.76#	0.76#	0.61	0.52	0.78#
Hodgkin lymphoma	0	0.79	0.84	0	3.65	0.43
Non-Hodgkin lymphoma	0	0.70#	0.71#	0.50	0.42	0.72#
Myeloma	0	0.83	0.80	1.01	1.66	0.72
Leukemia	1.53	0.81	0.85	0.58	0.00#	0.92

*SIR*, standardized incidence ratio, *RT*: radiotherapy, *BC*: bladder cancer, *SPC*: second primary cancer,

*KC*: kidney cancer, # significant at alpha = 0.05

At 21 years follow-up, 22,036 of the 48,875 BC patients had died. The 10-year overall survival (OS) rates were 46.2 and 52.6% in the SPC and non-SPC groups, respectively (*p* = 0.000). The 5- and 15-year OS rates for the SPC group were 72.3 and 28.3%, respectively, whereas those for the non-SPC group were 64.8 and 43.8%, respectively.

**Fig. 1:**
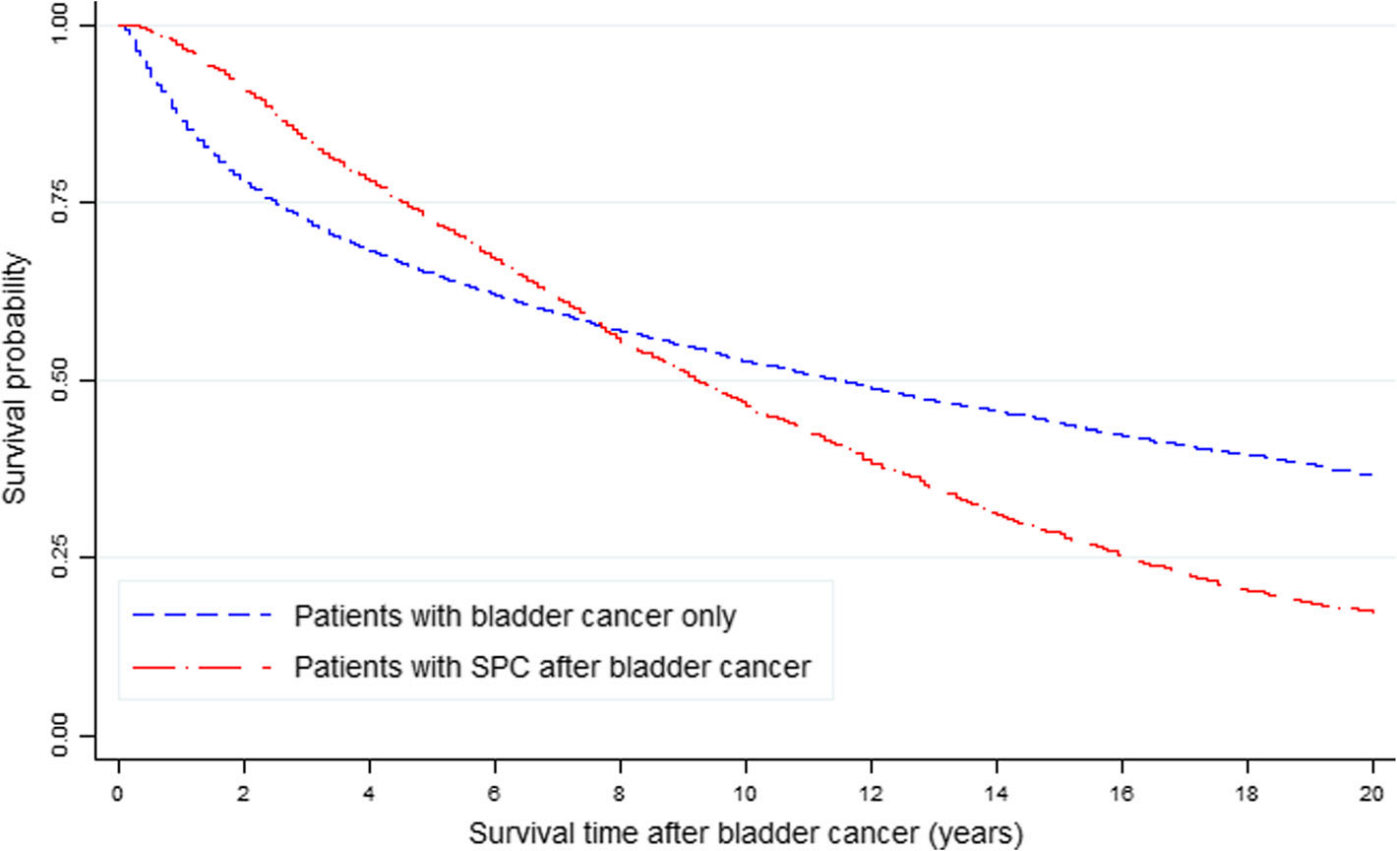
Kaplan-Meier curve: survival after bladder cancer according to the incidence of second primary cancer (SPC) in all patients

**Fig. 2:**
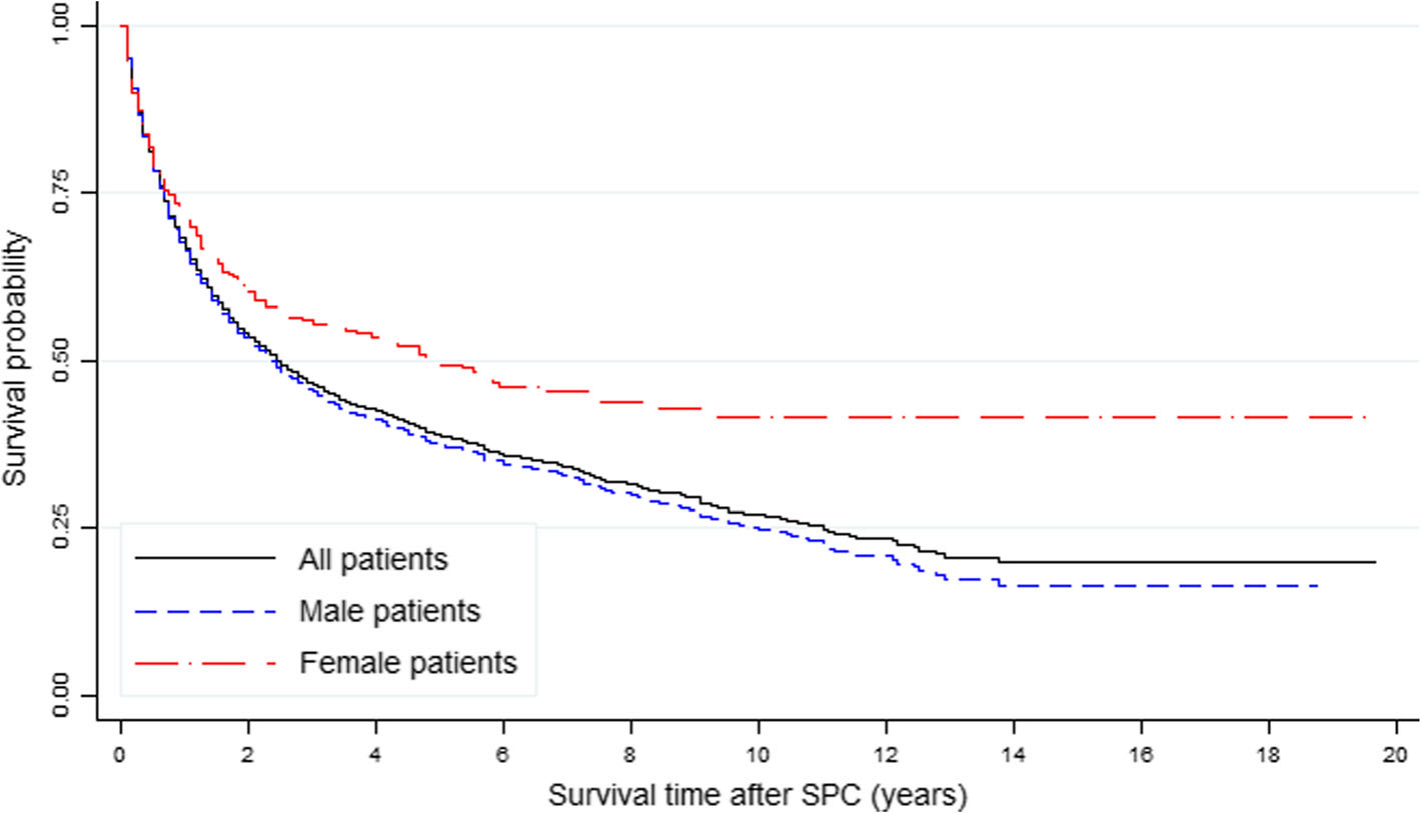
Kaplan-Meier curve: survival after second cancer according to sex in patients with second cancers

The survival curves crossed over time. The SPC group had higher OS rates compared with the non-SPC group for the first 8 years, but the OS of the SPC group declined thereafter (Fig. 1). After the onset of SPC, women had higher OS rates compared with men (Fig. 2).

We conducted a subgroup analysis of the patients treated between 2006 and 2013 to analyze any correlations between the SPC incidence and OS according to the SEER stage which were collected since 2006. After BC diagnosis, the OS curves of patients with SPC and non-SPC group crossed at 2.5 years (Additional file 1: Figure S1). Moreover, distant staging in SPC and non-SPC groups was estimated at 2.32 and 4.04% of cases, respectively. For patients with a follow-up of < 2.5 years, the proportion of distant staging was 5.31% at diagnosis, whereas that in those followed-up for ≥2.5 years it was 0.8% (*p* = 0.000). Before 2.5 years, the presence of SPC accounted for 5.38% of distant stage cases in the non-SPC group, and 3.45% of cases in the SPC group (*p* = 0.183). Afterwards, the proportions of distant staging were 0.48% in SPC and 0.82% in non-SPC group (*p* = 0.719) (Additional file 2: Figure S2).

## Discussion

SIRs for cancers that developed after primary BC were calculated using KCCR data. Analysis of the data revealed that, in the present cohort, BC survivors had a 6% lower risk of developing a new malignancy compared with the general population. Cancers of the tongue, tonsils, digestive system (e.g., stomach, colon, and liver) and non-Hodgkin lymphoma were less likely to occur as SPCs in patients with BC. However, these findings were incongruent with those reported previously [4, 5, 11]. While the reasons for reduced SPC risk in the BC patients are unclear, they might be related to smoking cessation and lifestyle modification after a BC diagnosis. Additionally, these results might, in part, be due to shared etiologies (genetic background and environment) and treatment-related factors [12].

A previous study that evaluated Korean patients with prostate cancer and kidney cancer using similar methods revealed SPC SIRs of 0.75 and 1.13, respectively [6, 7]. In the present study, the incidences of prostate cancer and kidney cancer were greater. This increased incidence might be due to shared etiological, environmental, and genetic factors between the first and second malignancies [13]. Moreover, a surveillance effect might contribute to increased risk immediately after diagnosis and might explain the elevated prostate cancer and kidney cancer risk after primary BC.

In a study examining associations between urinary tract cancers, Kinoshita et al. demonstrated that BC and prostate cancer share similar traits such as DNA repair and N-acetyl transferase polymorphism [14]. Kellen et al. reported that prostate cancer risk increases in patients < 70 years old within one year of BC diagnosis [15]. Lococo et al. also reported a significant increase in the relative risk of kidney cancer following BC [16].

In this study, we interestingly found that the risk for tongue and tonsil cancer significantly decreased in patients with BC, and the result for tongue cancer is significantly lower in those over 60 compared to those aged 40–59. Chemical factors like tobacco and alcohol, biological factors like human papillomavirus (HPV), syphilis, oro-dental factors, dietary deficiencies, chronic candidiasis and viruses have been known to be significantly associated with oral cancer [17]. The mechanism of the declined risk of tongue and tonsil cancer is still unclear. However, we speculated that life style modification (smoking cessation, diet, and so on) may reduce chance of developing tongue and tonsil cancer.

Smoking is a well-known risk factor for BC, kidney, lung, mouth, and pharynx cancers [18] and has been estimated to cause half of all BC cases in Western countries [19]. In contrast to our hypothesis, the present study did not show an increase in the number of subsequent respiratory system malignancies. Therefore, a sub analysis according to histological lung cancer type was performed, showing a significant increase in the incidence of lung squamous cell carcinoma and adenocarcinoma as SPCs. Possible reasons include potential etiological or genetic background differences between the Western and Asian patients, decreased smoking contribution compared with the West, and the possibility that the other cancers were smoking-related and occurred before the BC diagnosis.

The risk of cancer caused by radiation follows the individual exposed to radiation and continues to increase throughout the individual’s lifetime [20]. Studies evaluating the risk of secondary cancers after radiation therapy for prostate cancer have shown mixed results [21–23]. Recent meta-analyses have shown that patients who received prostate cancer radiotherapy are more likely to have a second malignancy of the bladder, colon and rectum than patients who have not received radiotherapy [24]. To our knowledge, although no studies have reported the risk of secondary cancer after radiation therapy for bladder cancer, our study showed that secondary cancers were more common in the digestive organs, such as the small intestine, colon, rectum, and female/male genital systems than patients who do not received radiotherapy. We assume that this result is related to the radiation field and is similar to the results of the meta-analysis mentioned above [24]. However, the lack of information such as the specific type of radiation treatment and dose of radiation is another limitation of this study.

To our knowledge, this is the first study to evaluate the histological subtypes of lung cancers as an SPC. Cigarette smoking is an established risk factor for lung cancer, but the severity of its association with other histologic types is unclear. Khuder [25, 26] reported that all histologic lung cancer types were significantly associated with cigarette smoking, and the association was stronger for squamous cell and small cell carcinomas compared with large cell cancer and adenocarcinoma. In the present study, squamous cell carcinoma and adenocarcinoma exhibited a significantly elevated risk of occurring as an SPC. Although not significant, small cell lung cancer risk also increased over an extended follow-up period. If smoking is a shared risk factor for BC and secondary lung cancer, the influence of smoking on each histologic type of secondary lung cancer in BC patients might be presumed to be different to that for primary lung cancer.

Cumulative survival curves of patients with or without SPC were estimated to investigate whether SPC affects the survival rate of patients who have BC. In particular, for the first 8 years, the SPC group had superior survival rates compared with the non-SPC group. Overall, this study demonstrated that patients in the non-SPC group had significantly more advanced BC at the time of diagnosis. Therefore, the survival rate of the non-SPC group was lower than that of the SPC group for the first 2.5 years after diagnosis of BC. Conversely, after 2.5 years the reverse was noted with survival in the SPC group being inferior to that in the non-SPC group. These findings suggested that the SPC group would require more attentive and systemic surveillance after 2.5 years of follow-up.

The present study has several limitations. First, information concerning several potential confounding variables including smoking, alcohol consumption, obesity, and familial cancer history were not available. Second, there was limited data available concerning genetic factors and specific cancer stages among the patients, making it impossible to evaluate the correlation between disease severity and SPC incidence. Third, the higher incidence of SPC might be associated with close surveillance or misclassifications because BC, prostate cancer, and kidney cancer often develop synchronously [15, 16]. Fourth, the median follow-up period was 4.13 years, which was not relatively long duration. Further studies with long follow-up periods will be needed to estimate the precise risk of developing SPC and to overcome surveillance bias. Fifth, it was impossible to divide into non-muscle invasive cancer and muscle invasive cancer in our study. The survival of patients with bladder cancer may be affected by degree of muscle invasion.

## Conclusion

The risk is lower among Korean survivors of BC compared to the expected risk of developing SPC in the general population. However, patients with BC remain at increased risk of some cancers, particularly prostate and kidney, lung squamous cell carcinoma, and lung adenocarcinoma. Therefore, longer and closer surveillance could be recommended for the early detection of SPC.

## Additional files

Additional file 1: **Figure S1.** Kaplan-Meier curve: survival after bladder cancer (BC) according to the incidence of a second primary cancer (SPC) in all patients (2006–2013). (TIF 1011 kb)

Additional file 2: **Figure S2.** Stage distributions by bladder cancer occurrence and follow-up years. (JPG 184 kb)

## Abbreviations

BC: Bladder cancer
CI: Confidence interval
KCCR: Korea Central Cancer Registry
RT: Radiotherapy
SD: Standard deviation
SEER: Surveillance epidemiology and end results
SIR: Standardized incidence ratio
SPC: Second primary cancer
