# Supplementary figures and images for: Risk of second primary Cancer among bladder Cancer patients: a population-based cohort study in Korea
Source: BMC Cancer. 2018 May 31;18:617. doi: 10.1186/s12885-018-4530-3 (PMC5984459; doi:10.1186/s12885-018-4530-3)

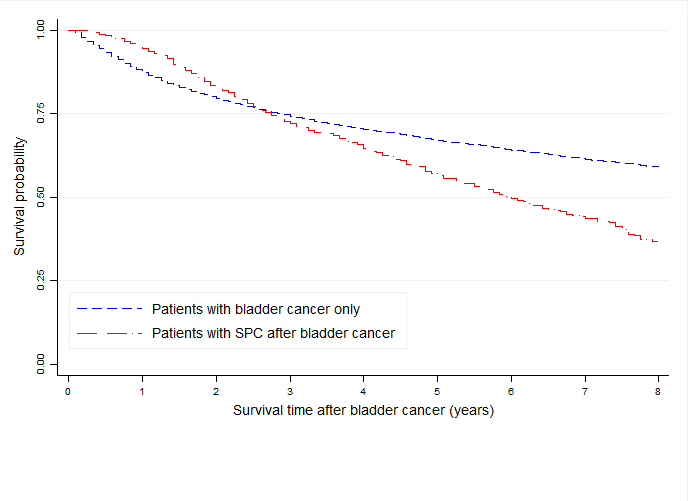

Supplement: Supplementary file 1 — Figure S1. Kaplan-Meier curve: survival after bladder cancer (BC) according to the incidence of a second primary cancer (SPC) in all patients (2006–2013). (TIF 1011 kb) [file 12885_2018_4530_MOESM1_ESM.tif]

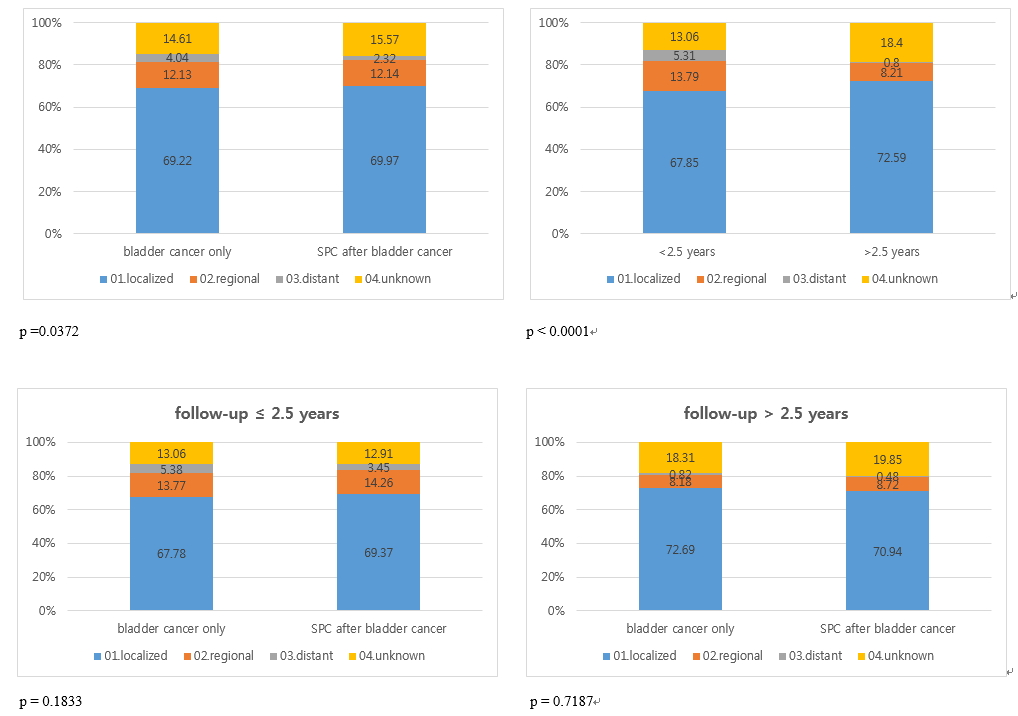

Supplement: Supplementary file 2 — Figure S2. Stage distributions by bladder cancer occurrence and follow-up years. (JPG 184 kb) [file 12885_2018_4530_MOESM2_ESM.jpg]
